# Which detergent is most suitable for the generation of an acellular pancreas bioscaffold?

**DOI:** 10.1590/1414-431X2024e13107

**Published:** 2024-08-19

**Authors:** M.C. Mantovani, N.R. Damaceno-Rodrigues, G.T.S. Ronatty, R.S. Segovia, C.A. Pantanali, V. Rocha-Santos, E.G. Caldini, M.C. Sogayar

**Affiliations:** 1 Grupo NUCEL de Terapia Celular e Molecular, Faculdade de Medicina, Universidade de São Paulo, São Paulo SP, Brasil; 2 Divisão Técnica de Apoio ao Ensino, Pesquisa e Inovação (DTAPEPI) - Centro de Biotecnologia e Inovação, Faculdade de Medicina, Universidade de São Paulo, São Paulo, SP, Brasil; 3 Departamento de Patologia, Laboratório de Biologia Celular, LIM 59, Faculdade de Medicina, Universidade de São Paulo, São Paulo, SP, Brasil; 4 Departamento de Gastroenterologia, Hospital das Clínicas, Faculdade de Medicina, Universidade de São Paulo, São Paulo, SP, Brasil; 5 Departamento de Bioquímica, Instituto de Química, Universidade de São Paulo, São Paulo, SP, Brasil

**Keywords:** Human acellular pancreatic matrix, Rat acellular pancreatic matrix, Sodium dodecyl sulfate (SDS), Sodium deoxycholate (SDC), Triton X-100, Pancreatic matrix recellularization

## Abstract

Pancreatic bioengineering is a potential therapeutic alternative for type 1 diabetes (T1D) in which the pancreas is decellularized, generating an acellular extracellular matrix (ECM) scaffold, which may be reconstituted by recellularization with several cell types to generate a bioartificial pancreas. No consensus for an ideal pancreatic decellularization protocol exists. Therefore, we aimed to determine the best-suited detergent by comparing sodium dodecyl sulfate (SDS), sodium deoxycholate (SDC), and Triton X-100 at different concentrations. Murine (n=12) and human pancreatic tissue from adult brain-dead donors (n=06) was harvested in accordance with Institutional Ethical Committee of the University of São Paulo Medical School (CEP-FMUSP) and decellularized under different detergent conditions. DNA content, histological analysis, and transmission and scanning electron microscopy were assessed. The most adequate condition for pancreatic decellularization was found to be 4% SDC, displaying: a) effective cell removal; b) maintenance of extracellular matrix architecture; c) proteoglycans, glycosaminoglycans (GAGs), and collagen fibers preservation. This protocol was extrapolated and successfully applied to human pancreas decellularization. The acellular ECM scaffold generated was recelullarized using human pancreatic islets primary clusters. 3D clusters were generated using 0.5×10^4^ cells and then placed on top of acellular pancreatic slices (25 and 50 μm thickness). These clusters tended to connect to the acellular matrix, with visible cells located in the periphery of the clusters interacting with the ECM network of the bioscaffold slices and continued to produce insulin. This study provided evidence on how to improve and accelerate the pancreas decellularization process, while maintaining its architecture and extracellular structure, aiming at pancreatic bioengineering.

## Introduction

Extracellular matrix (ECM) is a non-cellular component present in all organs and tissues, not only providing essential physical support for cellular constituents, but also mediating important biochemical and biomechanical stimuli, which are necessary for morphogenesis, cell differentiation, and tissue/organ homeostasis (1). ECM components participate in various cellular events, such as adhesion, migration, proliferation, cellular differentiation, apoptosis, angiogenesis, and cellular signaling, through storage and presentation of peptide growth factors, cytokines, and other soluble signaling molecules (1,2). The main components of the ECM are fibrillar (fibrillar collagens, laminin, fibronectin, and elastin), non-fibrillar (proteoglycans and glycosaminoglycans), microfibrils (non-fibrillar collagens and elastin associated microfibrils), and matrix recycling enzymes (metalloproteins, cathepsins, and heparinase) (2). In the pancreatic islets, the ECM is distributed throughout extra- and intracellular regions, associated to their permeable microvasculature, with the presence of type I, III, IV, and VI collagens and the predominance of type IV, fibronectin, laminin, and vitronectin, present in developing tissue but absent in mature tissue (3).

A biological skeleton (bioscaffold) of suitable material, containing biologically active molecules (3D bioscaffolds) play a critical role in Regenerative Medicine. Ideally, the bioscaffold provides a microenvironmental niche equal or similar to the native tissue (4). ECM influences cell proliferation, differentiation, and chemotaxis, inducing constructive tissue remodeling (5,6). Three-dimensional ultrastructure, surface topology, and extracellular matrix composition appear to be the main factors that contribute to these effects (7).

Decellularization involves the complete removal of cells and cellular debris from the tissue or organ, resulting in an acellular, non-immunogenic scaffold (8). The decellularization technique has been progressively applied to tissues of varying complexity, including highly intricate organs such as the pancreas (9). Several recent studies have shown that the pancreas can be decellularized, becoming an ECM bioscaffold, which can then be used as a basis for adhesion of several cell types to reconstitute the pancreas, with the aim of developing a bioartificial pancreatic tissue (8-14). Moreover, some studies have shown an increase in pancreatic islet functionality when these cells are cultured in decellularized matrices (8,15,16). In addition, pancreatic islet cell encapsulation platforms using ECM components support both functional survival of encapsulated islet grafts (17) and cell viability and differentiation, while significantly improving insulin delivery (18).

Decellularization methods usually combine physical and chemical treatments. Physical treatments may include agitation and sonication, mechanical action or pressure, and freezing and thawing. These methods rupture the cellular membrane and release the intracellular content. Enzymatic treatment, such as with trypsin, and chemical treatment, such as the use of ionic detergent solutions, rupture the cellular membrane and the bonds responsible for intra- and extracellular connections (19). The choice of the best decellularizing agent for tissues and organs depends on some factors, including tissue cellularity, density, lipid content, and thickness. It is necessary to bear in mind that every agent and decellularization method may alter the extracellular matrix composition and cause some degree of disruption to its ultrastructure (19).

For pancreatic decellularization, the literature describes different protocols employing mainly three types of detergent and/or their mixture, among which are: sodium dodecyl sulfate (SDS; 9,11,20,21), sodium deoxycholate (SDC; 8,22-24), and Triton X-100 (9-13,15,18,20,22,25-27). The methods available in the literature vary widely among different authors and a gold-standard method for pancreatic decellularization has not yet been found.

Using the method described for intestine decellularization that utilizes retrograde perfusion (28), our group developed a protocol to test the three detergents for pancreatic decellularization. Our approach can be applied to both rat and human pancreas, enabling a more rapid generation of decellularized biological scaffolds compared to other protocols described in the literature, which deserve further study as potential tools in Regenerative Medicine.

## Material and Methods

### Rat and human decellularized/acellular pancreatic matrix (bioscaffold)

The pancreas of male Wistar rats (n=12) weighing 300-500 g were harvested in accordance with the Institutional Ethical Committee for Animal Use of the University of São Paulo Medical School (CEUA-FMUSP; Protocol: 117/2015, approval date 15 July 2015). Human pancreases (n=6) from adult brain-dead donors were harvested in accordance with Institutional Ethical Committee of the University of São Paulo Medical School (CEP-FMUSP; CAAE Number: 47887115.6.0000.0065, approval code: 2.695.463, approval date 6 June 2018). Our group developed a protocol based on the description of intestine decellularization that utilizes retrograde perfusion (28). Murine pancreases were cannulated through the pancreatic Wirsung duct and the splenic vein pathways. Human pancreases were cannulated through the Wirsung duct and the splenic vein and artery pathways. Because this region is often injured during the pancreas resection process, with perforation of the local pancreatic capsule, the decision was made to remove the head portion of the organ and perform cannulation through three routes (pancreatic duct, splenic artery, and splenic vein). The size of the catheters used for cannulation in both the rat and human pancreases was suitable for the size of the duct and vessels, ranging from 14G (2.1×45 mm), 16G (1.70×45 mm), to 18G (1.30×45 mm), ensuring a perfect cannulation, with no slack. Continuous perfusion through the cannulated vessels was carried out using a peristaltic pump at 0.6 mL/min (for rat pancreas) and 1.5 mL/min (for human pancreas), initially with type I water, then with the detergent-enzymatic solution. For the rat pancreas, the detergent-enzymatic solutions were evaluated varying only the detergent - 1% SDS, 4% SDC, or 1% Triton X-100 - in association with DNase-I at 2000 kU. For the human pancreas, the detergent-enzymatic solution adopted was 4% SDC. At the end of the protocol, an extra wash with type I water was also adopted, summing up to a total of 31 h of perfusion for rat pancreas and 5 days for human pancreas. After this process, connective tissue and fat were removed. Bioscaffolds generated by decellularization were immediately used or maintained in saline phosphate buffer (PBSA) at 4°C until ready to use.

### DNA analysis

In order to compare the efficiency of different detergents in cellular removal, total genomic DNA quantification of the samples was carried out using wet tissue. For genomic DNA extraction and purification, the Illustra^TM^ Tissue & Cells GenomicPrep MiniSpin Kit (GE, USA) was used according to the manufacturer's specifications. DNA concentration and purity were estimated by spectrophotometric readings at 260/280 nm in the NanoDrop-1000 spectrophotometer (Thermo Fisher Scientific, USA).

### Histological analysis

Samples were fixed in 4% paraformaldehyde solution for 24 h and maintained in 70% alcohol. After fixation, the samples were dehydrated through incubation in increasing alcohol concentrations (70 to 100%), clarified in xylene, and then embedded in paraffin. Histological slices (4-5-μm thickness) were obtained using a microtome and then deposited onto silanized slides. Histological sections were deparaffinized and hydrated. For hematoxylin and eosin (H/E) staining, slides were stained with Harris hematoxylin for 1 min and 30 s, washed in running water for 2 min, stained with eosin for 12 s, and then washed in running water. For Picrosirius red staining, slides were stained with Picrosirius for 1 h, washed in running water for 3 min, stained with Harris hematoxylin for 1 min and 30 s, and then washed in running water for 2 min. For Alcian blue staining, slides were maintained in 3% acetic acid aqueous solution (pH 2.5) for 3 min and stained with Alcian blue for 2 h. The sections were washed again with 3% acetic acid for 3 min. For toluidine blue staining, slides were stained with toluidine blue for 1 h, washed in running water for 2 min, stained with eosin for 12 s, and then washed in running water. For resorcin-fuchsin staining with oxidation in oxone, slides were incubated in 10% oxone for 40 min, washed in running water for 5 min, and placed in 70% and then in 95% alcohol. Resorcin-fuchsin staining was carried out for 1 h. The samples were immersed in water to remove excess dye and differentiated in 70% alcohol for 8 min. All slides were dehydrated in baths with increasing concentrations of alcohol (70 to 100%), cleared in xylene, mounted with histological glue, covered with coverslips, and maintained at room temperature until evaluation using an Optiphot-2 Nikon optical microscope, with a Nikon Digital Sight DS-U1 camera and the NIS Elements Nikon^®^ program (Japan).

### Transmission electron microscopy (TEM)

Biopsies (3-5-mm long, 1-mm wide, and 1-mm thick) were collected for ultrastructural analysis, fixed in 2% glutaraldehyde solution in 0.15 M PBSA at pH 7.2 and 0.1% tannic acid, and maintained at 4°C for 2 h. Samples were then conditioned in 5% glucose saline solution, washed in 5% glucose saline solution, incubated in osmium tetroxide at 4°C for 2 h, further washed in 5% glucose saline solution, and then incubated in uranyl acetate at 4°C overnight. For the resin blocking, the material was previously dehydrated in increasing acetone concentrations (30 to 100%), washed in propylene oxide solution, and incubated for 3 h in propylene oxide solution and resin in equal proportions at room temperature. Samples were then incubated in pure resin at 37°C for 1 h, inserted into the inclusion molds with pure resin and maintained at 60°C for 72 h. Semi-thin sections (300-nm thickness) obtained using a microtome (Leica^®^, Germany) were placed onto glass slides, stained with 1% methylene blue solution for 1 min at room temperature, and washed in distilled water. The ultra-thin sections (70 nanometers thickness) were obtained using a microtome (Leica^®^) and mounted onto a copper plate. Staining was carried out by addition of a drop of lead citrate solution over the sections and incubation for 1 min at room temperature. The generated images were captured through a transmission electron microscope (JEOL JEM.1010 Electron Microscope, Japan).

### Scanning electron microscopy (SEM)

Samples collected for SEM were fixed in 2.5% glutaraldehyde solution in 0.1 M PBSA (pH 7.4) for 1 h, washed (3×) in 0.1M PBSA for 15 min each, and then incubated in 1% osmium tetroxide at 4°C for 1 h followed by washing (3×) in PBSA for 15 min each, dehydration in increasing alcohol concentrations (50 to 100%) previously filtered with a 0.22 μm filter, dried in K850 Critical Point Dryer (Quorum Technologies Ltd., UK), collected onto copper conductive adhesive tape (3M, USA) and covered with gold on the Desk II Sputter Coater (Denton Vacuum, USA) using 180-s exposures. SEM images were obtained using a LEO 435VP microscope (Carl Zeiss, Germany).

### Isolation and 2D culture of human pancreatic islets

The pancreas of an adult brain-dead human donor (n=1) was harvested in accordance with Brazilian regulations and the Institutional ethical committee of the University of São Paulo Medical School (CEP-FMUSP; CAAE number: 47887115.6.0000.0065, approval code: 2.695.463, approval date 6 June 2018) for pancreatic islets isolation and processing. Pancreatic islets were isolated after ductal distension of the pancreas and tissue digestion with collagenase and neutral protease (SERVA Electrophoresis, Germany), according to the automated method of Ricordi (27) with modifications (28), as previously described (29). Briefly, islet purification was achieved using a continuous Ficoll density gradient in a COBE 2991 Cell Processor (Gambro/Terumo BCT, Japan). The islet preparation used in this study exhibited a 70±4% purity, as determined by dithizone staining, and greater than 80% islet cell viability, which was evaluated using the live/dead fluorescent method, based on incorporation of either fluorescein diacetate (Sigma-Aldrich, USA) by live cells or propidium iodide (Sigma-Aldrich, USA) by dead cells. Upon isolation, the islets (2×10^4^ islet equivalents IEQ/100 cm^2^ plate) were maintained in adherent cultures in CMRL 1066 medium (5.6 mM glucose) (Mediatech-Cellgro, USA), supplemented with 1 mM L-glutamine (Sigma-Aldrich), 0.2% ciprofloxacin (Sigma-Aldrich, USA), and 10% fetal calf serum (FCS) (Cultilab, Campinas, Brazil) at 37°C with 5% CO_2_, as previously described (30).

### Human primary pancreatic islet cell-derived 3D cultures

The 3D cultures of cells derived from the two-dimensional (2D) primary human pancreatic islet adherent cultures were generated using the NanoShuttle™ Kit (Greiner Bio-One, Germany) 3D magnetic culture method, according to the manufacturer's specifications. Briefly, the cultured islet cells were removed from the adherent culture plate surface and, once in suspension, the cells were impregnated by centrifugation 3× (400 X *g*, 5 min, room temperature) in the presence of the NanoShuttle™ (Greiner Bio-One, Germany) PL reagent, consisting of gold, iron oxide, and poly-L-lysine nanoparticles. As described by the manufacturer, this reagent remains fixed to the cell membrane for up to eight days, at which point cells are released to the culture medium, being biocompatible and having no effects on metabolism, cell proliferation, or inflammatory stress. After ligation of the nanoparticles, 0.5×10^4^ cells were plated onto a non-adherent culture plate, which was placed on top of the magnetic base (or the magnetic accessory), which centralizes the cells and promotes their interaction to form 3D structures (spheroids), in the presence of CMRL 1066 medium (5.6 mM glucose) (Mediatech-Cellgro), supplemented with 1 mM L-glutamine (Sigma-Aldrich), 0.2% ciprofloxacin (Sigma-Aldrich), and 10% fetal calf serum (FCS) (Cultilab), and maintained at 37°C and 5% CO_2_, with medium replacement every three days.

### Human decellularized/acellular pancreatic matrix (bioscaffold) slices

To generate thin sections, samples of approximately 1 mm long, 1 mm wide, and 1 mm thick were included in Tissue-Tek O.C.T. Compound (Optimal Cutting Temperature, Sakura, Japan) and maintained frozen at -80°C until the moment of use. The samples were transported on dry ice to the cryostat (Microm GmbH, Germany) to generate histological sections of 25 and 50 μm. The slices were collected on coverslips, placed onto 24-well non-adherent culture plates and maintained in PBSA at 4°C until ready to use.

### Insulin assay

The 3D primary cultures of human pancreatic islets were cultured under standard conditions for 48h and then subjected to insulin secretion in response to glucose stimulation. Krebs-Henseleit solutions (Sigma-Aldrich) supplemented with 0.2% albumin (Griffols Brasil, Brazil) and containing low (2.8 mM) or high (16.7 mM) glucose concentrations were used. For this assay, five spheroids were selected after discarding the cell culture supernatant and incubated for 1 h at 37°C in a solution of low (2.8 mM) glucose concentration. Subsequently, cells were washed three times with PBSA and incubated for another 1h at 37°C in a solution also of low (2.8 mM) glucose concentration. The culture supernatants obtained were stored at -20°C. The procedure was repeated, but the cells were then incubated for 1h at 37°C in a solution of high (16.7 mM) glucose concentration. The incubated solutions were also stored at -20°C for subsequent analysis of the insulin secreted by the cells. The samples were assayed using enzyme-linked immunosorbent assays (ELISA), using commercial kits for insulin detection (ELISA kit, Millipore, USA), according to the manufacturer's instructions.

Supernatants from 3D cell culture spheroids of human primary pancreatic islets in contact with the pancreatic extracellular matrix of 25- and 50-μm slices were subjected to insulin quantification using the Insulin Quantitative Reagent Kit (Elecsys Insulin, Roche^®^, USA) according to the manufacturer's specifications in the automated Cobas E 601^®^ equipment (Roche^®^).

### Statistical analysis

Data are reported as means±SD. The statistical differences between group means were determined by ANOVA followed by Tukey's *post hoc* test or by unpaired two-tailed *t*-test after confirming the normal data distribution. P<0.05 was considered as statistically significant.

## Results

### Rat pancreas decellularization

**Figure 1:**
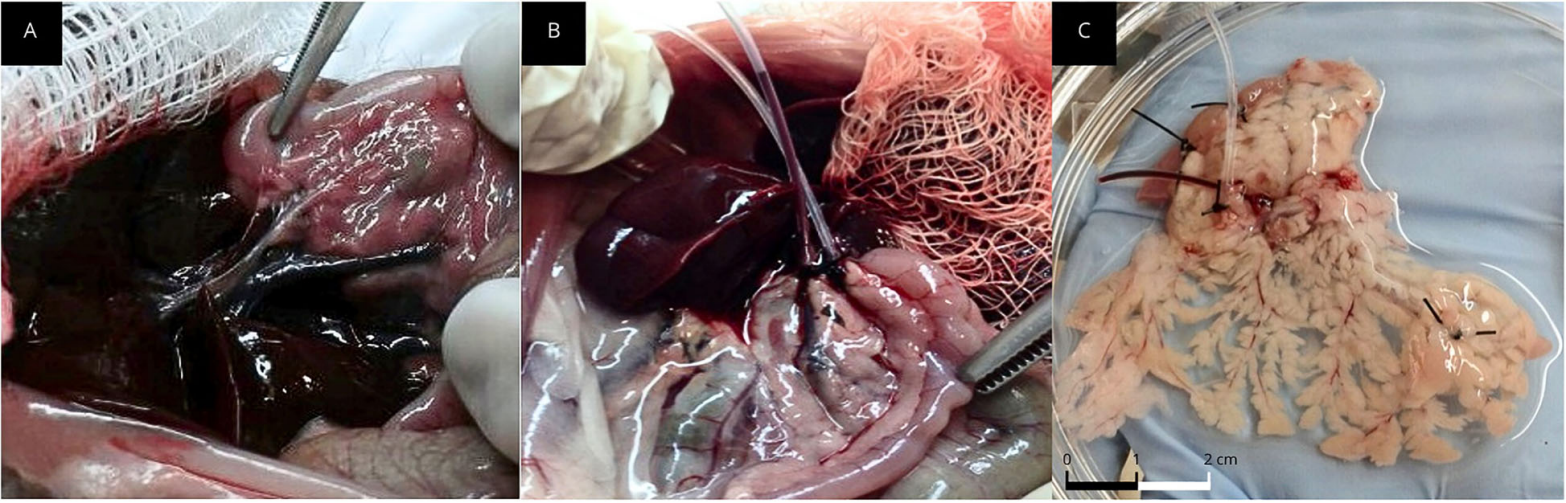
Murine pancreas cannulation and removal. The rat pancreas was exposed, and the pancreatic duct and portal vein branch were visualized (**A**) and cannulated (**B**). **C**, Detail of rat pancreatic tissue after cannulation, showing the organ's diffuse anatomy in a standard Petri dish. The black and and white scale bars are each 1 cm.

We standardized the cannulation, extraction, and decellularization techniques of the rat pancreas. The pancreas of each animal was exposed and the pancreatic duct and portal vein branch were cannulated, after which the pancreas was removed as a block together with the spleen and part of the intestine and maintained in PBSA at 4°C until processing (Figure 1).

For decellularization by retrograde perfusion through the above-mentioned vessels, three different detergent-enzymatic solutions were used, namely: 1% SDS or 4% SDC or 1% Triton X-100 for a total period of 31h using the same protocol that was developed by our group based on the description of intestine decellularization that utilizes retrograde perfusion (28), changing only the detergent.

**Figure 2:**
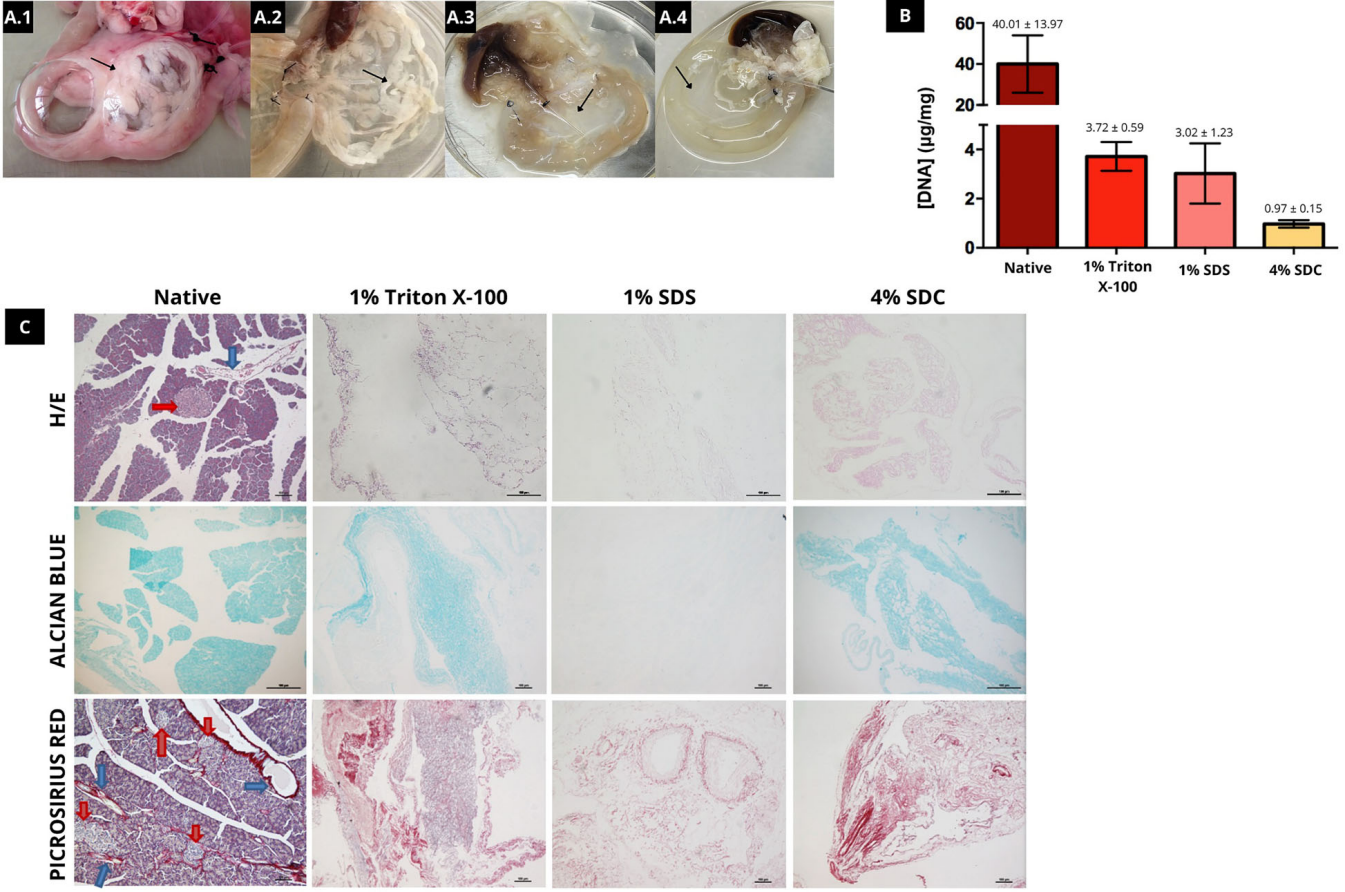
Evaluation of different detergents. **A**, Macroscopic evaluation. **A.1**, Native rat pancreas. **A.2**, Rat pancreas decellularized with 1% Triton X-100 detergent. **A.3**, Rat pancreas decellularized with 1% sodium dodecyl sulfate (SDS) detergent. **A.4**, Rat pancreas decellularized with 4% sodium deoxycholate (SDC) detergent. Arrows indicate the pancreas. **B**, Genomic DNA quantification of native rat pancreas samples and rat pancreas decellularized with 1% Triton X-100, 1% SDS and 4% SDC; (n=3). **C**, Histology of native rat pancreas and rat pancreas decellularized with 1% Triton X-100, 1% SDS, and 4% SDC detergents using different staining agents. Red arrows indicate pancreatic islets and blue arrows indicate blood vessels. Scale bars indicate 100 μm.

Upon macroscopic analysis of the pancreas after decellularization with different detergents, 1% SDS proved to be the most aggressive to the pancreatic extracellular matrix, as evidenced by the loss of characteristic scaffold architecture, which was not observed when employing 1% Triton X-100. However, this detergent proved to be inefficient in completely removing cellular content. In contrast, the application of 4% SDC provided, in principle, the best result, proving to be efficient in cellular removal while maintaining macroscopic scaffold architecture (Figure 2A).

As shown in Figure 2B, the amount of DNA remaining in the wet pancreas tissue subjected to decellularization protocols was clearly less than that present in the native tissue, namely: native tissue 40.01±13.97 ng/mg, 1% Triton X-100 3.72±0.59 ng/mg, 1% SDS 3.02±1.23 ng/mg, and 4% SDC 0.97±0.15 ng/mg, but no statistically significant difference was found between the detergents employed.

As shown in Figure 2C, staining with HE indicated that tissue decellularized with 1% Triton X-100 maintained the extracellular matrix architecture, but some cells remained. With 1% SDS, no cells remained, but the extracellular matrix integrity was not maintained and regions other than those next to blood vessels and ducts were stained. Conversely, no cells remained in tissue decellularized with 4% SDC and the integrity of native extracellular matrix architecture was maintained. Alcian blue staining indicated remaining proteoglycans and GAGs. Of all the decellularized scaffolds, however, those resulting from 1% SDS decellularization presented decreased staining. Picrosirius red staining showed that all the decellularized tissues maintained their collagen scaffold. Moreover, apparent and noteworthy rupture of collagen fibers was observed in several areas of scaffolds upon 1% SDS treatment (Figure 2C).

**Figure 3:**
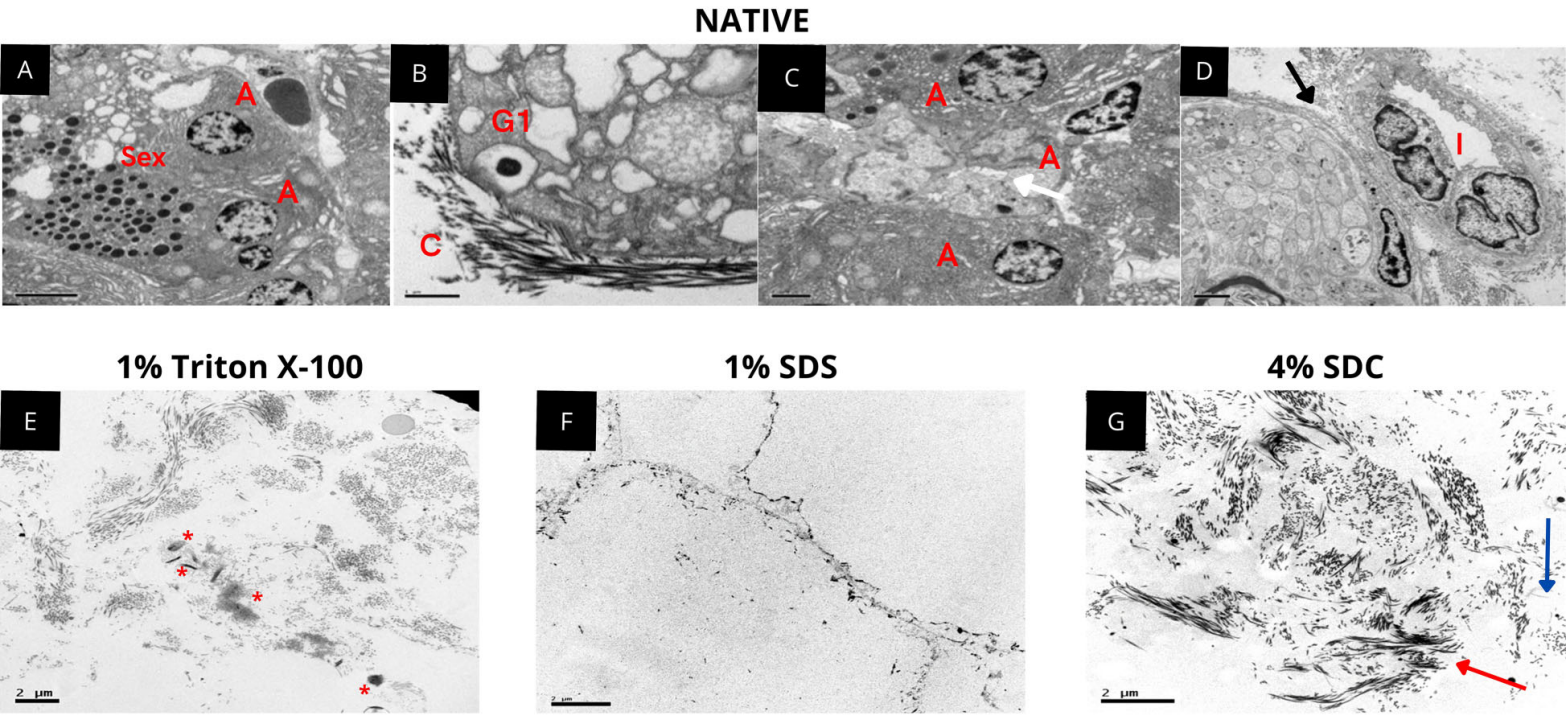
Evaluation of different detergents by transmission electron microscopy. **A**, **B**, **C**, and **D**, Rat native pancreatic tissue with details indicating: Sex: Exocrine secretion;. A: Acini; GI: Insulin granules; C: Collagen; I: Pancreatic islet. The black arrow indicates the pancreatic duct. The white arrow indicates a blood vessel. **E**, Rat pancreas decellularized with 1% Triton X-100 detergent. Asterisks indicate cellular remains. **F**, Rat pancreas decellularized with 1% sodium dodecyl sulfate (SDS) detergent. **G**, Rat pancreas decellularized with 4% sodium deoxycholate (SDC) detergent. The red arrow indicates proteoglycans and glycosaminoglycans. The blue arrow indicates collagen fibers/fibrils. Scale bars indicate 2 μm.

Transmission electron microscopy (TEM) was employed to compare the ultrastructure of decellularized scaffolds (Figure 3), indicating that tissue decellularized with 1% Triton X-100 maintained the extracellular matrix architecture, preserving collagen fibers and fibrils, proteoglycans, and GAGs, but cellular residues were observed adhered to the scaffold. In tissue decellularized with 1% SDS, a large loss of characteristic tissue architecture was observed, with ruptures in collagen fibers and fibrils and areas in which these structures were absent. It was not possible to detect proteoglycans and GAGs in the analyzed samples. Conversely, in pancreatic tissue decellularized with 4% SDC, no remaining cells were observed, and the tissue ultrastructure was preserved, with the presence of collagen fibers and fibrils, as well as proteoglycans and GAGs (Figure 3).

### Human pancreas decellularization

**Figure 4:**
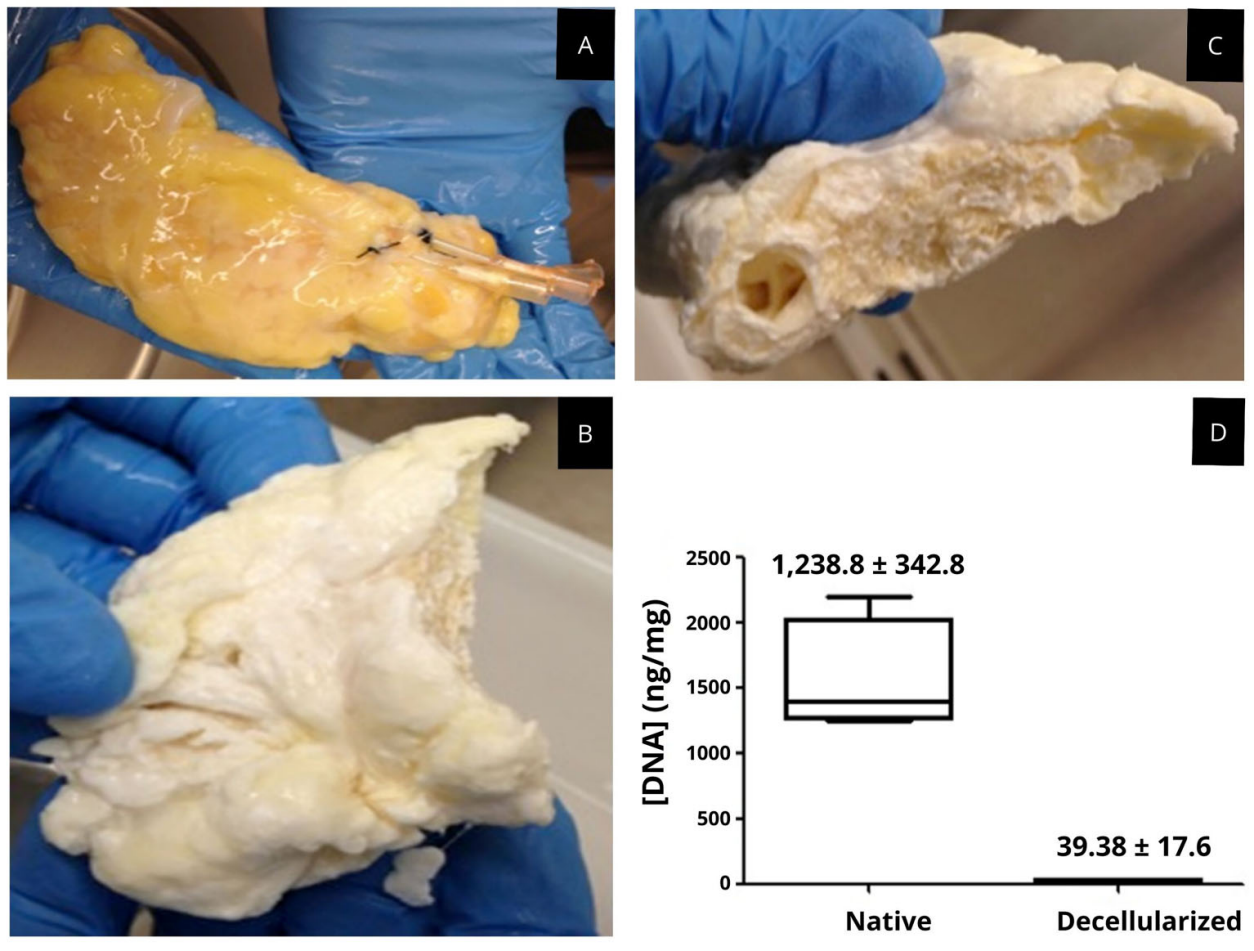
Macroscopic view of human pancreas. **A**, Native human pancreas. **B**, Decellularized human pancreas; whitened and spongy external portion of the scaffold is shown. **C**, Decellularized human pancreas; whitened and spongy internal portion of the scaffold is shown. **D**, DNA quantification. Quantification of genomic DNA in native pancreas and decellularized human pancreas samples (n=6). Data are reported as median and interquartile range.

The initial attempts at cannulation and subsequent perfusion of the human pancreas for decellularization were carried out through two routes (pancreatic duct or Wirsung duct and splenic artery). Evaluations indicated the presence of DNA and residual cells (data not shown), mainly in the region of the pancreas head. The decision to remove the head portion of the organ and perform cannulation through three routes (pancreatic duct, splenic artery, and splenic vein) was made. Decellularized pancreases are shown in Figure 4A-C. The remaining amount of DNA observed in human wet pancreatic tissue subjected to the decellularization protocol was less than that present in native tissue, namely: native tissue 1.238.8±342.8 ng/mg (P<0.0001) and decellularized scaffold 39.38±17.6 ng/mg, demonstrating that approximately 97% of human pancreatic DNA was removed (Figure 4D).

**Figure 5:**
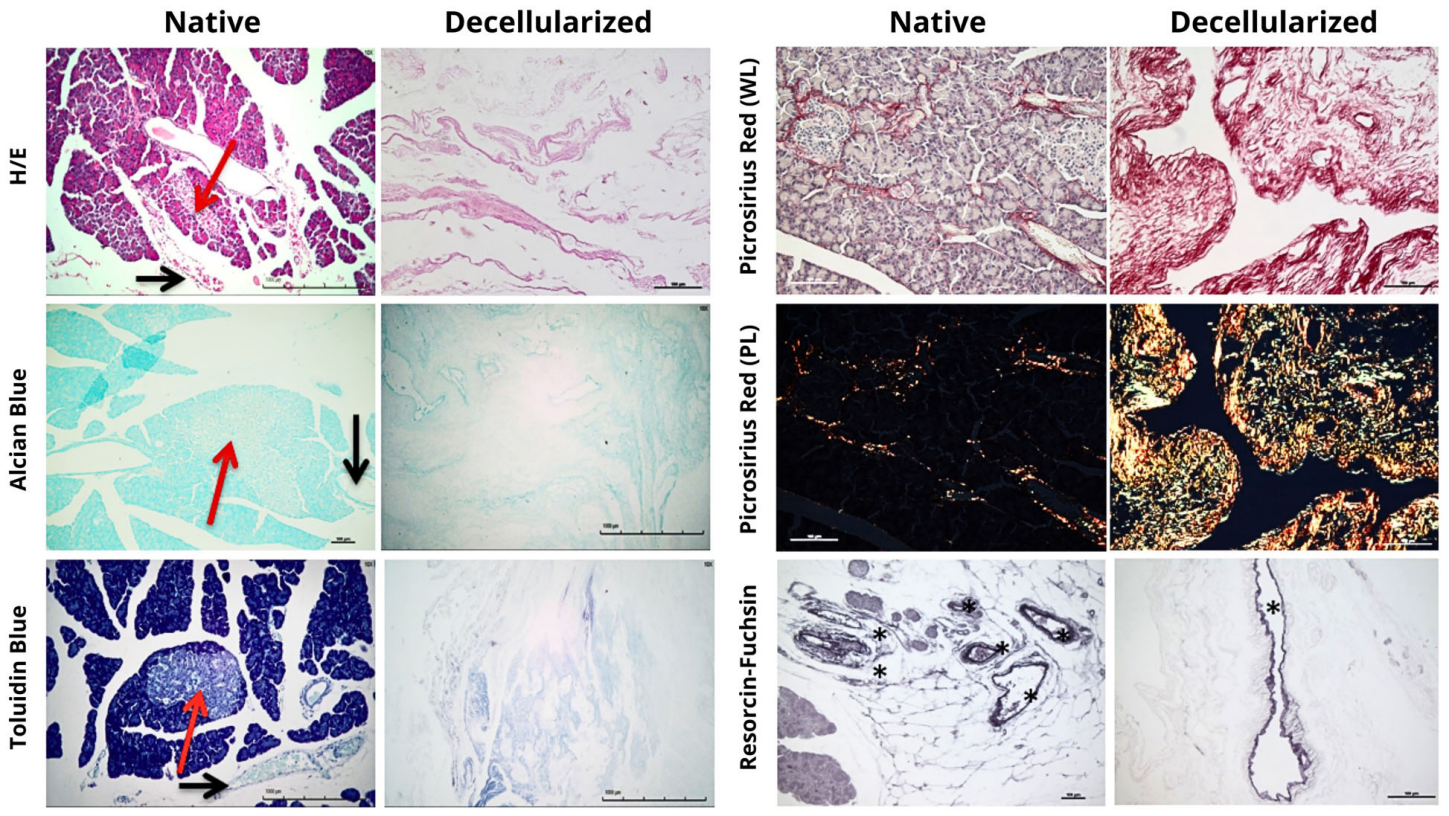
Histology of the human pancreas. Native and decellularized human pancreas stained with H/E, Alcian blue, toluidine blue, Picrosirius red (WL: white light; PL: polarized light), and Resorcin-Fuchsin with oxidation in oxone staining. Red arrows indicate pancreatic islets and black arrows indicate blood vessels. Asterisks indicate elastic fibers. Scale bars indicate 100 μm.

Qualitatively, HE staining showed that the decellularized human pancreatic tissue also did not present remaining cells and maintained the integrity of the native extracellular matrix architecture. Alcian blue and toluidine blue staining revealed proteoglycans and GAGs remains in all human decellularized scaffolds. Picrosirius red staining showed that all decellularized human tissue also maintained collagen scaffolds, preserving thin fibers, as well as intermediate and thick fibers, identified using both white and polarized light. Resorcin-fuchsin staining, with oxidation using oxone, revealed maintenance of elastic fibers in human tissue upon decellularization (Figure 5).

**Figure 6:**
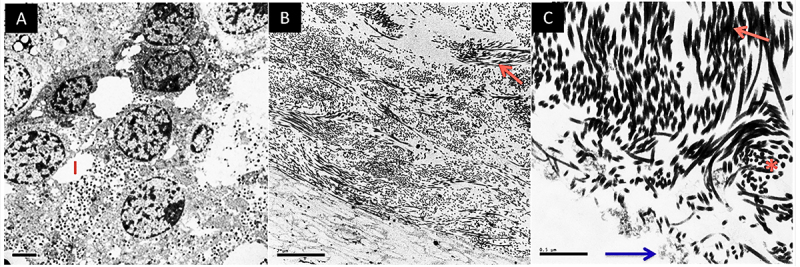
Ultrastructure by transmission electron microscopy of the human pancreas. **A**, Native human pancreas. Scale bar indicates 2 μm. **B**, Decellularized human pancreas. Scale bar indicates 2 μm. **C**, Detail of decellularized scaffold indicating collagen strands and matrix proteoglycans and glycosaminoglycans. Scale bar indicates 0.5 μm. The blue arrow indicates matrix proteoglycans and glycosaminoglycans and red arrows indicate collagen strands. I: Pancreatic islet; *transversal section of collagen strands.

The ultrastructure of decellularized human scaffolds evaluated through TEM indicated the presence of cellular nuclei and pancreatic islets in native tissue, as well as insulin granules and exocrine secretions, collagen fibers, and fibrils and the presence of proteoglycans and GAGs. In all decellularized scaffold samples, no cells or cellular remains were observed, displaying collagen fibers/fibrils, as well as proteoglycans and GAGs (Figure 6).

**Figure 7:**
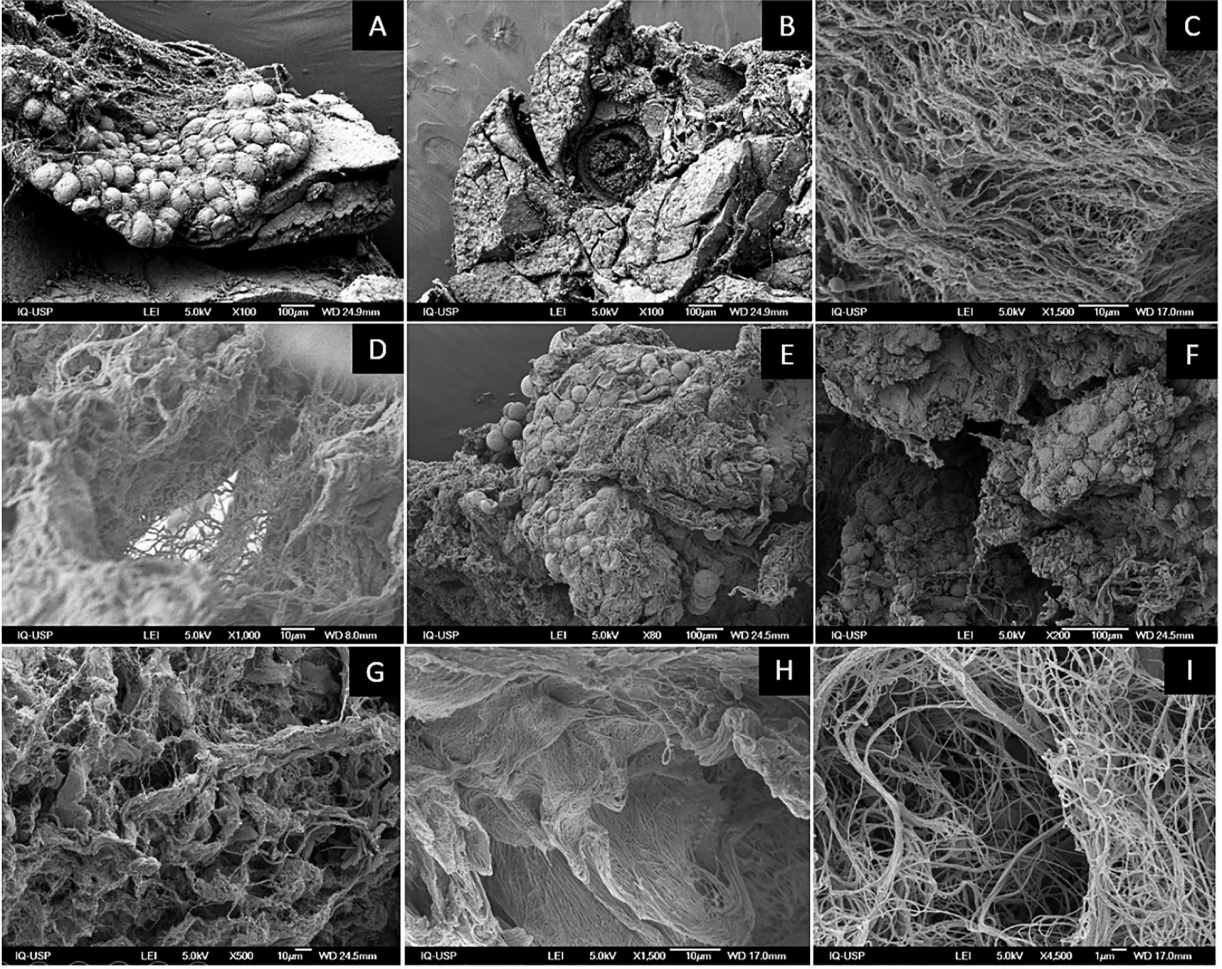
Native and decellularized rat and human pancreases by scanning electron microscopy. **A** and **B**, Native rat pancreas. Scale bar indicates 100 μm. **C**, Decellularized rat bioscaffold. Scale bar indicates 10 μm. **D**, Decellularized rat scaffold. Scale bar indicates 10 μm. **E** and **F**, Native human pancreas. Scale bar indicates 100 μm. **G**, Decellularized human scaffold within thicker collagen fibers. Scale bar indicates 10 μm. **H**, Decellularized human scaffold within thinner collagen fibers. Scale bar indicates 10 μm. **I**, Decellularized human scaffold within thinner collagen fibers. Scale bar indicates 1 μm.

SEM was also used to evaluate the surface of native and decellularized (with 4% SDC) murine and human pancreas, qualitatively revealing the efficiency of our decellularization protocol and allowing the comparison of the generated scaffold architectures. In both murine and human native tissue, organized and compartmentalized cellular structures, with the presence of blood vessels and collagen fibers/fibrils were observed. Cells and cellular residues were not observed, but only collagen strands with gaps previously occupied by cells, with thicker and thinner fibers in the generated human scaffolds (Figure 7).

### Recellularization

**Figure 8:**
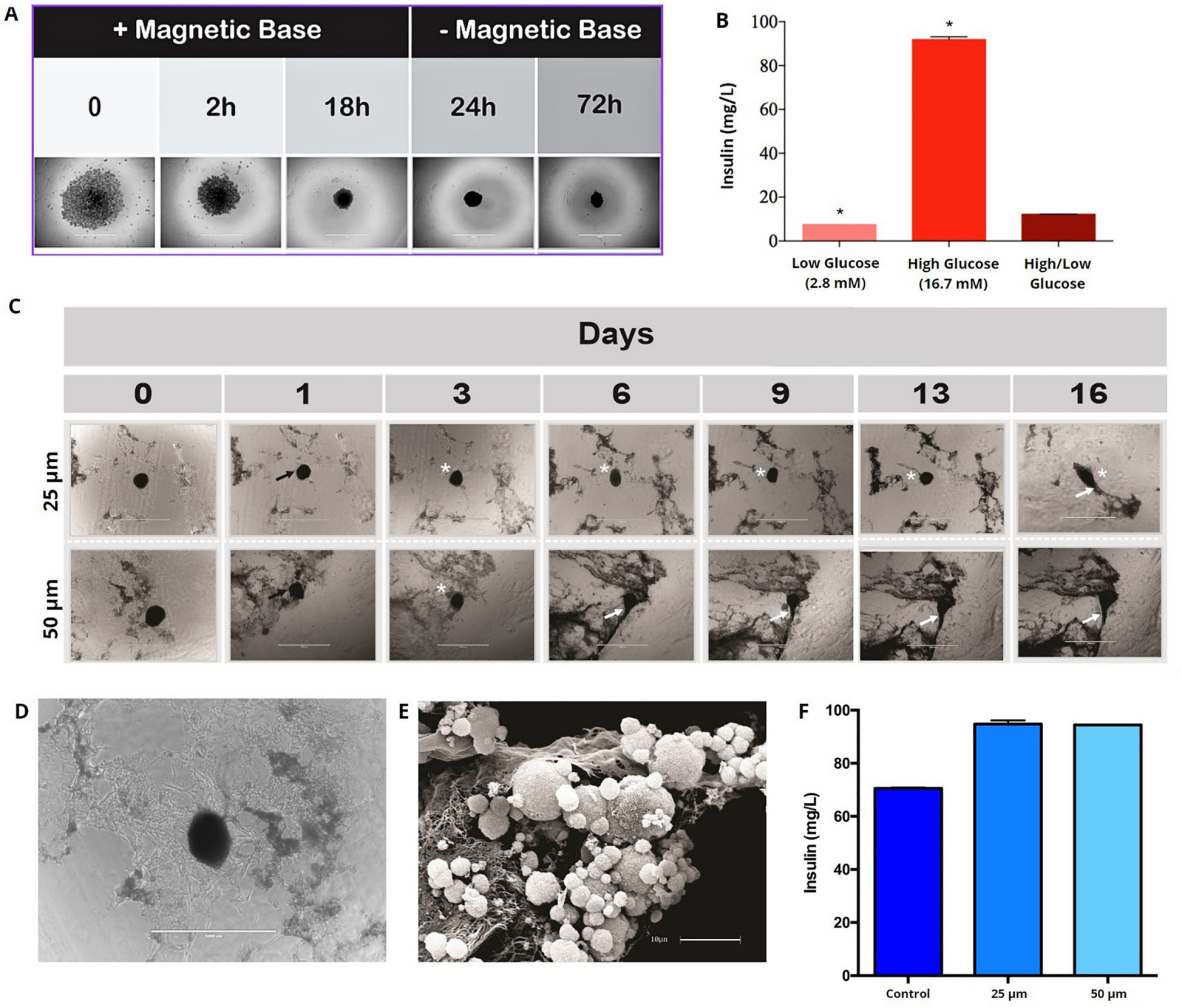
**A**, Human primary pancreatic islet cell-derived 3D cultures (cell clusters) plated onto human decellularized pancreatic scaffold, first with a magnetic base for 0, 2, and 18 h and secondly without the magnetic base for additional 24 and 72 h. Scale bar indicates 1000 μm. **B**, Insulin secretion (mg/L). **C**, 3D cultured clustered cells generated from 0.5×10^4^ cells were placed onto human decellularized pancreatic scaffold slices (25 and 50 μm) observed at 0, 1, 3, 6, 9, 13, and 16 days. The black arrows indicate cell clusters association with the bioscaffold. The white asterisks indicate cells adhered to the scaffold. The white arrows indicate clusters folded in with the scaffold. The same cluster was monitored at different time points. Scale bars indicate 1000 μm. **D**, Detail of a spheroid in the presence of the decellularized pancreatic scaffold indicating the presence of cells adhered to the bioscaffold slice. Scale bar indicates 1000 μm. **E**, SEM of human primary pancreatic islets cell-derived 3D cell clusters plated onto a human acellular pancreatic matrix. Three-dimensional view revealed the nanofibrous structures of the human decellularized pancreatic scaffold and their interaction with the cell clusters with visible cells outside the cluster. Scale bar indicates 10 μm. **F**, Insulin release (mg/L). These assays were performed using the culture medium collected at the 16th day after the human primary pancreatic islet-derived 3D clusters were cultured onto 25 and 50 μm decellularized pancreatic scaffold slices. Control: 3D cluster alone. Data are reported as means and SD. *P<0.05, compared to control.

To verify whether the pancreatic acellular extracellular matrix bioscaffold could be recellularized, we evaluated the interaction between the human primary pancreatic islet cell-derived 3D cultures and the acellular pancreatic matrix. Dissociated primary human islet cells from adherent 2D cultures may be reaggregated into 3D cell clusters, allowing the control of the cell amount, cell types, and cell cluster interactions with the pancreatic bioscaffold. The generation of the primary pancreatic islet cell-derived 3D-culture using a magnetic reagent was carried out in a remarkably short period of time (only 18 h), with complete cluster formation (Figure 8A) and insulin secretion in response to glucose (Figure 8B). The spheroids originated from 0.5×10^4^ cells/well were placed onto acellular pancreatic slices of 25 and 50 μm thickness. They attached on the first days after plating (days 1 to 3), began to proliferate between the 3rd and the 6th day, and thereafter tended to fold and intertwine with the scaffold (Figure 8C-E), maintaining insulin secretion and release (Figure 8F).

## Discussion

In the initial pancreatic decellularization studies, sections of mouse pancreas (8) or whole pancreas from mice (9), pigs (15), and humans (12) were decellularized using different detergents or their combinations (SDS (9), SDC (8), and Triton X-100 (9,12,15) in distinct preliminary protocols. After a few years, the literature still describes different protocols for pancreatic decellularization, employing mainly three types of detergents and/or their mixtures, namely: SDS (11,20,21), SDC (22-24), and Triton X-100 (10,11,13,18,20-22,25-27).

In the absence of consensus in the literature, we decided to evaluate the best-suited detergent using a protocol previously employed for whole-organ pancreatic decellularization. Our group developed a protocol based on the description of intestine decellularization that utilizes retrograde perfusion (28), varying only the detergents employed. The experiments were initiated with rat pancreas, hoping to extrapolate the methodology to human pancreas.

It has been described in literature that a variable that may affect the mechanical properties of decellularized scaffolds is the duration of exposure to decellularizing agents, as demonstrated by a protocol for tracheal decellularization, which involved repeated cycles of SDC and DNase, evidencing profound structural changes between cycles 18 and 22, rendering the decellularized scaffold inadequate due to mechanical alterations (29).

In our protocol, all detergent treatments resulted in some remaining DNA content, although within the acceptable residue limit described in the literature for decellularized tissues (19). Upon macroscopic and microscopic evaluation of the scaffolds obtained, 1% SDS proved to be the most aggressive agent to the pancreatic extracellular matrix, as evidenced by the loss of its characteristic scaffold architecture, with apparent rupture of collagen fibers and low staining of proteoglycans and GAGs. With 1% Triton X-100, the scaffold architecture was maintained, although it proved inefficient for total cellular removal, since remaining cells and cellular debris were observed. Lastly, with 4% SDC, efficient cellular removal was observed, with maintenance of the pancreatic scaffold architecture.

Conflicting reports are found in the literature on the effects of SDS and Triton X-100 on the properties of various decellularized tissues, even those with similar characteristics, including rupture of collagen fibers, amount of GAGs removed, and effects on the viscoelastic behavior of scaffolds (19).

SDS is an anionic surfactant, which is effective in disrupting hydrophilic and lipophilic interactions and solubilizing cell membranes. It possesses a strongly polarized sulfate head group and a linear alkyl tail. During decellularization, SDS has the capacity to completely remove cellular tissue, nucleic remnants, and cytoplasmic proteins. However, it is damaging to structural and signaling proteins and not effective at maintaining GAGs, growth factors or collagen fibers, and the ultrastructure, being best employed in conjunction with milder surfactants (30).

SDC is another anionic surfactant derived from deoxycholic acid, a secondary bile acid that can disrupt membranes. Despite being anionic, much of its micellization properties are due to the rigid and concave hydrophobic portion of the molecule, which allows for different aggregation numbers, depending on the concentration and polar or non-polar substrate with which it interacts (31). Known from as early as 1917 for its potency in dissolving *Streptococcus pneumoniae bacterium* cultures (32), its efficacy as a decellularizing surfactant was first demonstrated in the isolation of kidney-derived basement membranes, wherein their ultrastructures were maintained, in contrast to other ultrasound treatments available at the time (33). Further work was pursued toward obtaining basement membranes of several tissues (34), and later on, extracellular matrices (35) and acellular grafts (36).

Triton X-100 is a synthetic non-ionic surfactant, being therefore less harsh than compounds such as SDS and SDC in damaging tissue integrity (37). It possesses an aromatic lipophilic group and a hydrophilic polyethylene oxide chain.

Reports by Hudson et al. (36) set the gold standard for using the now discontinued Triton X-200 anionic surfactant for obtaining acellular nerve grafts. To replace it, protocols using Triton X-100 (38) and SDC (39) in conjunction with enzymes have been employed, which yielded similar decellularization profiles. With respect to current whole-organ decellularization, the efficacy of detergents will vary depending on the target tissue's cellularity, density, lipid content, and thickness (30). Different surfactants can be used individually or in combination to fine-tune the protocol to specific needs.

Based on our results, the condition that proved to be the most adequate for rat pancreatic decellularization was 4% SDC. Our rat pancreas decellularization protocol was performed in a period of 31h, a contrasting time interval compared to that previously described for murine (144 h) (9) and porcine (168 h) (15) pancreas.

Furthermore, it is important to highlight that this detergent has recently been approved in the USA by the Food and Drug Administration (FDA) for human use in esthetic application/injections for the treatment of submentonian adiposity (popularly known as “double chin”), by Kythera Biopharmaceuticals Inc., Protocol No. 206333 (https://www.accessdata.fda.gov/drugsatfda_SDCs/nda/2015/206333Orig1s000TOC.cfm). This approval sets a precedent, expediting future applications of SDC in the production of human decellularized scaffolds for the generation of a bioartificial pancreas.

To verify whether the methodology for decellularization of murine pancreas using 4% SDC may be extrapolated and applied to human pancreas decellularization, human pancreases were decellularized using the same protocol, but for a longer period of time in the final wash with type I water (5 days). The initial attempts at cannulation and subsequent perfusion of the human pancreas for decellularization were carried out through two routes (pancreatic duct or Wirsung duct and splenic artery). Subsequent evaluation indicated the presence of residual DNA and cells (data not shown), mainly in the region of the pancreas head. This region, which contains the pancreatic duct, is predominantly irrigated by another artery (superior mesenteric artery). Due to the fact that this region is often injured during the pancreas resection process, with perforation of the local pancreatic capsule, a decision was made to remove the head portion of the organ and perform cannulation through three routes (pancreatic duct, splenic artery, and splenic vein). This led to better results and the standardization of the protocol for human pancreas decellularization. Peloso et al. (12) performed cannulation and subsequent perfusion through the pancreatic duct, superior mesenteric artery, and splenic artery, obtaining a scaffold with a different macroscopic aspect, when compared to the native organ. The human pancreatic scaffolds that we generated maintained the macroscopic spongy and white aspect, while those shown in Peloso et al. (12) are pale yellow in color, but their histological evaluation of ultrastructure and DNA content is similar to ours. Our protocol proved to be efficient in cell removal, as judged by the residual genomic DNA quantification in samples, with values being compatible with those reported in the literature for decellularized tissue (<50 ng/mg of remaining dry-weight DNA) (19). Moreover, these acellular scaffolds can be recellularized by 3D human pancreatic primary cell clusters, which tend to rapidly associate with the acellular matrix, showing visible cells in the outermost regions of the spheres and displaying positive insulin secretion.

In summary, decellularization with 4% SDC generated scaffolds that did not present remaining cells, while preserving the architecture of the native extracellular matrix, revealing thick, intermediate, and thin collagen fibers, and maintaining proteoglycans and GAGs in the matrix, showing that both murine and human pancreas may be effectively decellularized using the protocol described in this study.

In Brazil, organ donation increased by 13% of potential donors (55 pmp) in 2021, the highest rate ever. However, the donation rate fell by 13%, due to a 24% decrease in actual donations. This means that until 2019, three potential donors were required for an effective donation, whereas currently four potential donors are required for the same result (40). Therefore, for several reasons, many organs that are discarded, including the pancreas, could be salvaged for use in the process of decellularization and generation of acellular pancreatic scaffolds.

### Conclusion

Whole-organ decellularization for generation of acellular scaffolds to be used in Regenerative Medicine has become a reality. Our results demonstrated that the most adequate detergent for pancreatic decellularization is 4% SDC, which may be applied not only to murine pancreas, but also for efficient decellularization of human pancreas, generating acellular scaffolds that can be recellularized by 3D human pancreatic primary cell clusters, showing association, visible cells in the outermost regions of the spheres, and insulin secretion. Interestingly, this detergent was recently approved by the FDA for use in humans in the esthetic treatment of submentonian adiposity, setting a precedent for possible future application of SDC for the generation of human decellularized pancreatic scaffolds aiming at creating a bioartificial pancreas. Therefore, the use of 4% SDC is very promising, since the method is practical and reproducible, and should be further explored to open new avenues for the production of decellularized scaffolds.
